# How Frequently Is Asthma Objectively Demonstrated before Starting a Biologic? Quality Assessment of a Group Practice of Allergists and Immunologists

**DOI:** 10.3390/ijerph17249482

**Published:** 2020-12-18

**Authors:** Iwona Dziewa, Timothy Craig, Taha Al-Shaikhly

**Affiliations:** 1 Section of Allergy and Immunology, Department of Medicine, Penn State College of Medicine, Hershey, PA 17033, USA; talshaikhly@pennstatehealth.psu.edu; 2 Section of Allergy and Immunology, Department of Medicine and Pediatrics, Penn State College of Medicine, Hershey, PA 17033, USA; tcraig@pennstatehealth.psu.edu

**Keywords:** severe asthma, diagnosis, biologic therapy, comorbidity, severe asthma checklist

## Abstract

Worldwide, asthma-related healthcare cost remains a major burden. Individuals with severe asthma account for 50% of that cost. Although they are expensive, biologics such as anti-IL5 and anti-IgE agents promise cost-effectiveness when judiciously used to decrease asthma-related hospitalization and the debilitating side effects of systemic corticosteroids. Before considering biologics to treat patients with asthma, current guidelines recommend confirmation of asthma and control of comorbid diseases. Diagnostic confirmation of asthma can be challenging among individuals with severe asthma. In this quality assessment study, we determined the frequency of objective asthma confirmation and addressing of comorbidities prior to starting biologics at a group practice of allergists and immunologists. We surveyed our specialty providers to understand habit(s) leading to the observed results. We identified 40 adult patients who started on biologic modifiers for asthma over the past 5 years. Only 58% of these patients had a proper diagnosis of asthma. Providers underutilized several diagnostic methods that may prove useful in confirming asthma diagnosis in this patient population. The factors contributing to poor asthma control were rarely addressed. A sense of urgency to initiate biologics was the primary reason for the observed results. Further interventions are needed to improve asthma diagnosis and management prior to the initiation of biologic therapeutics.

## 1. Introduction

Asthma, a chronic inflammatory disease of the airways [1], remains among the most common chronic diseases in all ages [2]. Its prevalence varies between 1–18% in different parts of the world [3]. In high income countries, asthma prevalence is as high as 15–20% [4]. In the United States, the prevalence of asthma during the period 2007–2017 ranged from 7.3–8.5% [5]. Asthma prevalence continues to rise throughout the world [6], and asthma confers a significant burden worldwide [7]. In the United States, from 2008 to 2013, the estimated total cost of asthma care was USD 81.9 billion [8]. This cost is projected to increase over the next 20 years, and adherence to evidence-based management of asthma has the potential to significantly decrease costs [9].

Asthma has been classified into phenotypes based on the clinical characteristics resulting from these factors [10]. Severe asthma is one of these phenotypes [11]. Recently, the term “endotype” has emerged and is used to classify asthma based on the molecular mechanisms contributing to the disease pathogenesis. The classification of asthma into endotypes has allowed for a personalized treatment approach [12]. The two main endotypes of asthma are labeled as type 2 (T2) high or T2-low [12]. In T2-high asthma, airway inflammation is driven by the release of interleukin-4 (IL-4), interleukin-5 (IL-5) and interleukin-13 (IL-13) [13]. These cytokines are released by cells of the immune system, predominantly the type-2 helper cell (Th2) [14,15]. T2-high asthma is more steroid responsive than T2-low asthma [15], which is driven by other immune effector cells such as T-helper 17 cells.

The currently available biologic treatments target the Th2 cytokines or T2-high asthma [12]. Clinical trials of biologic modifiers that block immunoglobulin (Ig) E, IL-5 or IL-4 receptor alpha (IL-4R alpha) subunit have demonstrated efficacy and safety in treating patients with uncontrolled severe asthma [16,17,18,19,20]. Presently, benralizumab, dupilumab mepolizumab, omalizumab, and reslizumab are approved by the US Food and Drug Administration (FDA) for the treatment of severe asthma with a T2-high endotype [21]. Severe asthmatics comprise 5–10% of the asthmatic population but are responsible for up to 50% of healthcare costs associated with asthma [22]. Biologic therapeutics are expensive. In the US, the annual wholesale acquisition cost can range from USD 30,889 to USD 39,048 per biologic agent [23]. For cost-effective use of these medications, careful selection of candidate patients is of paramount importance. Biologic therapy should be considered for patients with severe asthma who remain uncontrolled despite maximum inhaler therapy consisting of at least an inhaled corticosteroid (ICS) plus another controller [3,24]. The most recent publication by the Global Initiative for Asthma (GINA) makes a distinction between difficult-to-treat asthma and severe asthma [3]. The guidelines advocate for the careful assessment of difficult-to-treat asthmatics by ensuring these patients have a correct diagnosis and adhere to the treatment plan and by ensuring their asthma comorbidities are properly addressed prior to labeling them with severe uncontrolled asthma and escalating therapy with the addition of biologic modifiers.

In the United States, asthma can be diagnosed by either a primary care provider or a specialist. A 2015 study showed that the use of spirometry within a year of a new asthma diagnosis averaged at 47%; spirometry was utilized the least in primary care (23.3%) and more frequently in specialty care (80.1%) [25]. Other studies have shown that asthma is over diagnosed in about one third of individuals [26,27]. However, the frequency of proper asthma diagnosis before starting this costly biologic treatment is not well studied. In this quality assessment, we examined the frequency of objective asthma diagnosis and assessment of asthma comorbidities prior to starting biologics in a group Allergy Immunology specialty practice with an ultimate goal of providing high value care. We hypothesize that asthma is not objectively confirmed in a significant number of patients because asthma diagnosis can be especially challenging in individuals with severe asthma who have already been started on ICS and long-acting bronchodilators that can confound current diagnostic modalities.

## 2. Materials and Methods

We conducted a retrospective chart review to examine compliance with the current GINA 2020 guidelines. Inclusion criteria were patients between the age of 18 and 80 years, who were seen at an Adult Allergy and Immunology Clinic staffed by many providers from 1 January 2015 to 30 August 2020 and received biologic therapeutics for asthma. All providers specialized in the field of Allergy and Immunology and consisted of doctors of medicine or osteopathy and one nurse practitioner. Exclusion criteria included the presence of other lung diseases such as interstitial lung diseases, alpha-1 antitrypsin deficiency and eosinophilic granulomatosis with polyangiitis, and the use of these biologics for other indications such as chronic idiopathic urticaria or atopic dermatitis. We selected our patient population by manually reviewing the electronic medical records (EMRs) of patients presented to the clinic to receive biologics, and we extracted relevant data. The accuracy of research data was ensured by random internal quality and assurance checking of the data. Data were collected by the co-investigator (I.D.) and randomly checked for accuracy of reporting by the principal investigator (T.A.-S.). The study met the criteria for exempt research as determined by the Human Subjects Protection Office at Penn State College of Medicine (Hershey, PA, USA).

We evaluated for objective confirmation of asthma prior to the initiation of biologics. As per the current GINA guidelines, asthma was considered confirmed if patients had any of the following: (1) flow volume loop with a positive bronchodilator response, (2) positive bronchial challenge test such as a methacholine challenge, (3) a significant increase in lung function after 4 weeks of anti-inflammatory treatment, (4) positive exercise challenge and/or mannitol hyperresponsiveness, (5) excessive variation in FEV_1_ (forced expiratory volume in the first second) in between visits and/or (6) excessive variability in twice-daily peak expiratory flow (PEF) over two weeks [3]. We checked for proper asthma diagnosis by examining spirometry tests and pulmonary function tests, bronchial challenges and by a chart search for “asthma confirmation”. We evaluated whether asthma treatment was optimized prior to starting biologics. We considered asthma medications optimized if patients were on a medium or high dose ICS-LABA and a long-acting muscarinic agent (LAMA) or medium or high dose ICS-LABA and a leukotriene inhibitor at the time the biologic was started [28]. We also examined whether comorbidities were addressed. Comorbidities examined included gastroesophageal reflux disease (GERD), vocal cord dysfunction, the use of beta-blockers or an angiotensin-converting enzyme inhibitors (ACE-Is), anxiety, depression, obesity, and proper inhaler technique. Examination was performed by a chart search of the words and phrases: “GERD, reflux, PPI, proton pump inhibitor, H2, vocal, cord, videostroboscopy, depression/depressed, anxiety/anxious, obesity/obese, inhaler, inhaler technique, beta-blocker, ace, inhibitor”, as well as manual examination of medication lists at the time the biologic agent was prescribed. After collecting the data, we sent an electronic survey to clinic providers (n = 10) with the aim to identify reasons for the results we obtained from the manual review of EMRs. The survey questions were made using the Likert scale. Survey data were collected and managed using REDCap electronic data capture tools [29,30].

Descriptive analyses were performed to determine the median and interquartile range for continuous variables and the number and relative frequency of categorical variables. Statistical significance between the different groups was assessed using non-parametric statistics including the Mann–Whitney U test for continuous data and Fisher’s exact test for categorical data. Analysis was performed using GraphPad Prism version 9 (GraphPad Software, San Diego, CA, USA).

## 3. Results

We identified 40 patients treated with biologics for asthma in the group practice. The baseline demographics are summarized in Table 1. The patients had a mean age of 56 years at the time of our review, and 26 patients (65%) were females. Omalizumab was started in 29 patients, benralizumab in 6 patients, mepolizumab in 4 patients and dupilumab in 1 patient. Almost all of these patients (95%) had their asthma medication optimized with the addition of non-biologic add on therapy of either a leukotriene receptor antagonist or a LAMA [28].

### 3.1. Asthma Confirmation

**Figure 1:**
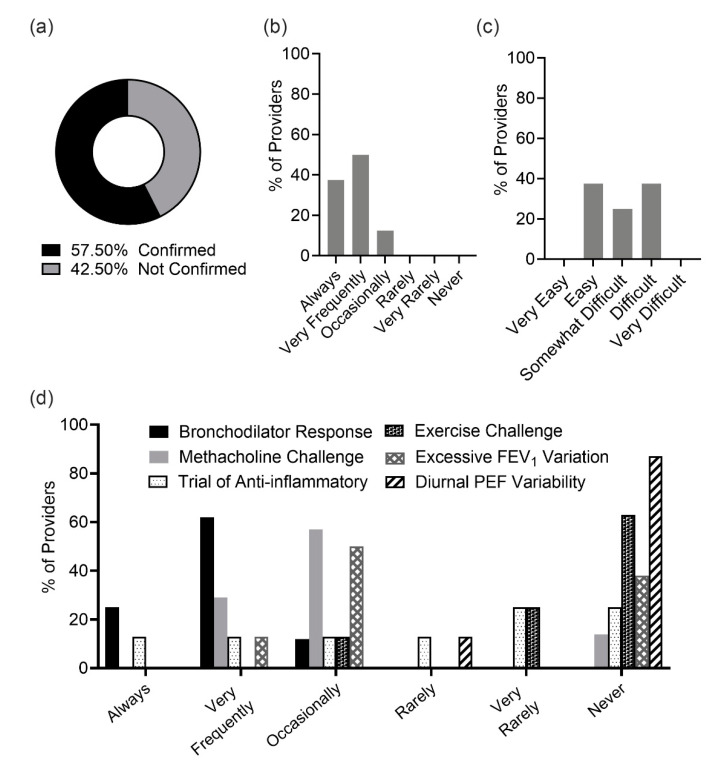
Asthma Confirmation: (**a**) The proportion of patients with an appropriate confirmatory test prior to initiation of biologic therapy, (**b**) shows survey results on how often providers seek to confirm asthma diagnosis prior to starting biologic modifiers, (**c**) shows survey results on provider perception on the difficulty of confirming asthma diagnosis, (**d**) shows providers’ response to a survey question on how often they use the various methods to confirm asthma diagnosis in their patients before starting biologic modifiers.

Evaluation of the EMR data showed that asthma was objectively confirmed in only 23 patients (58%) (Figure 1a). The median age of patients with confirmed asthma diagnosis was similar to those without confirmed asthma diagnosis (55 versus 58, *p* = 0.56). Asthma was equally confirmed among female and male patients (46% versus 79%, *p* = 0.09), and among obese and non-obese individuals (55% versus 64%, *p* = 0.65). However, the median FEV_1_ % predicted for patients with unconfirmed asthma was higher than those with confirmed asthma (72 versus 58, *p* = 0.036). Allergy providers who responded to our survey (n = 8) reported that they objectively confirm asthma: always (37.5%), very frequently (50%) or occasionally (12.5%) (Figure 1b). Survey data showed that 37.5% of providers considered confirming asthma diagnosis easy, 25% somewhat difficult and 37.5% difficult (Figure 1c). Providers who reported difficulty in confirming asthma diagnosis prior to starting biologic modifiers indicated that “there is a sense of urgency to start biologics” as the primary reason behind not confirming asthma diagnosis (60%), while another 40% indicated that “patients with severe asthma often have airway remodeling and tests are generally unreliable”. Another 40% stated that “stepping down or up on asthma medication and re-evaluating FEV_1_ is not usually feasible as patients are uncontrolled”.

The EMR data showed that out of the 23 patients whose asthma was confirmed, 22 (96%) had their asthma diagnosis confirmed by a positive bronchodilator reversibility test and 1 patient (4.3%) had a confirmation of asthma diagnosis by demonstration of airway hyperresponsiveness to mannitol. This prompted us to survey the providers’ habits on their commonly used methods for asthma confirmation. The survey results are shown in Figure 1d. The most frequent method of asthma diagnosis reported by providers was flow volume loop with bronchodilator response assessment, which was reported as being used always or very frequently by 87.5% of our providers. The second most commonly used method was demonstration of airway hyperresponsiveness with a methacholine challenge, which was reported as being used very frequently or occasionally by 85.7% of providers. Another method reported by providers as chosen often was excessive variation in FEV_1_ in between visits, which was used very frequently by 12.5% and occasionally by 50%. The remaining methods were not used often, including demonstrating a significant increase in FEV_1_ after 4 weeks of anti-inflammatory treatment, the evaluation of indirect hyperresponsiveness by an exercise challenge and/or mannitol, and demonstrating excessive variability in twice-daily PEF over two weeks. Demonstrating a significant increase in lung function after 4 weeks of anti-inflammatory treatment was rarely, very rarely or never used by 62.5% of our providers as reported in the survey, while an exercise challenge and/or mannitol hyperresponsiveness was used very rarely or never used (87.5%). All providers either rarely (12.5%) or never (87.5%) confirmed asthma by demonstrating excessive variability in twice-daily PEF over two weeks. The measurement of fractional exhaled nitric oxide (FeNO) was used by 25% of the providers to confirm asthma diagnosis.

### 3.2. Asthma Comorbidities

As summarized in Table 1, the review of the EMR data showed that GERD was evaluated in 28 clinic patients (70%). Either a prior diagnosis of vocal cord dysfunction or an evaluation of vocal cord dysfunction was performed in 10 patients (25%). A beta-blocker or an ACE-I were taken by 10 patients (25%), and none of these patients (0%) had their medication use addressed by counseling. Either a pre-existing diagnosis of depression or anxiety or evaluation for these conditions was performed in 18 patients (45%). The average BMI of all patients was 35.2 kg/m^2^ and 29 patients (73%) had obesity, defined as having a BMI of ≥30 kg/m^2^; however, obesity was evaluated in only 4 patients (14%). Lastly, proper inhaler technique was reviewed in 9 patients (22.5%).

**Figure 2:**
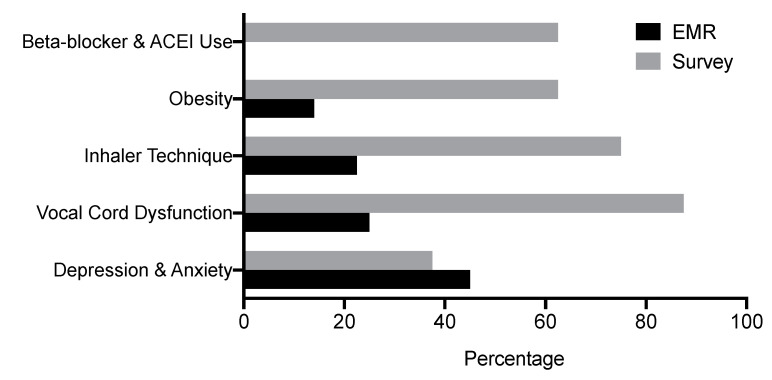
Asthma Comorbidities. The frequency of comorbidities addressed in a clinic based on a review of EMR data compared to the percentage of providers who reported addressing these comorbidities very frequently or always. The survey results showed a discrepancy between the EMR data and providers’ perception of their performance.

These results prompted us to survey providers on their habits of addressing these comorbidities. We surveyed providers on whether they review inhaler technique and address obesity, anxiety, depression, vocal cord dysfunction and the use of beta-blockers and ACE-Is. Since GERD was fairly well-addressed in our clinic, we did not question our providers on this topic. In the survey, 87.5% of providers reported addressing vocal cord dysfunction very frequently. Inhaler technique was reported as “reviewed” either always or very frequently by 75% of our providers, while 62.5% of providers reported very frequently addressing the use of beta-blockers or ACE-Is. The majority of the providers (62.5%) reported that they address obesity always or very frequently. The most common reason stated for failing to address obesity was “there is often a sense of urgency to start biologics” (100%). Some providers (37.5%) reported very frequently addressing anxiety and depression. The most common reason for not addressing anxiety and depression was “failing to remember to do so” (80%). The survey results showed a discrepancy between the EMR data and providers’ perception of their performance (Figure 2).

## 4. Discussion

Managing patients with severe asthma remains challenging. The difficulty can start with the first step, which is proper confirmation of asthma diagnosis. Additionally, in many patients with difficult-to-control asthma, other factors may contribute to poor control, including GERD, vocal cord dysfunction, the use of beta-blockers or ACE-Is, anxiety, depression, obesity, or proper inhaler technique [3,31,32]. Before escalating therapy with the addition of biologics in patients whose disease is uncontrolled, current GINA 2020 guidelines recommend proper asthma diagnosis and thorough evaluation to exclude masquerading diseases and contributory factors. Once asthma is objectively confirmed and contributory factors are addressed, patients with “difficult-to-control asthma” are thence classified as having “severe asthma,” and these patients may benefit from the use of biologic modifiers. Therefore, this thorough evaluation can prove cost-effective by eliminating the cost associated with the unnecessary use of biologic treatment.

Our study showed that an unusually high proportion of patients (42.5%) lacked objective confirmation of their asthma diagnosis prior to the initiation of biologic treatment. This figure is slightly higher than the rate of physician-diagnosed asthma in a prospective, multicenter cohort study [27]. In that study, asthma diagnosis was not established in 33.1% of patients. However, in contrast to our study, it included patients with both mild and severe asthma. This higher proportion of patients who were started on biologics without confirmation of asthma diagnosis suggests that asthma diagnosis may be even more difficult in severe asthmatics. This is further supported by the self-reported rate of asthma confirmation via the electronic survey, which was much higher compared to the EMR data we collected, implying that providers do aim to confirm asthma diagnosis but may find it difficult to do so. Further, our survey highlighted that providers were pressured by a sense of urgency because of the instability of asthma when approaching these patients, which might have interfered with asthma confirmation. Providers in our study underutilized several diagnostic methods that may prove useful in confirming asthma diagnosis in this patient population, and cited airway remodeling and a sense of urgency as reasons for not pursuing confirmatory studies. Almost all patients (96%) who had a proper confirmation of their diagnosis had their asthma confirmed by a positive bronchodilator reversibility test, which is defined as an increase in FEV_1_ by 12% and 200 mL after administration of 200–400 mcg of rapid-acting bronchodilator [3]. Demonstrating direct airway hyperresponsiveness (e.g., a methacholine challenge) was the second most utilized method. While demonstrating a positive bronchodilator response is specific for asthma diagnosis (specificity, 90–93%), it has a limited sensitivity of 13–29% [33,34]. The sensitivity of bronchodilator response assessment can be complicated by the use of anti-inflammatory and bronchodilator therapies. A methacholine challenge is 69–97% sensitive and 57–78% specific, and is regarded as the test of choice for ruling out asthma [33,35,36]. However, airway hyperresponsiveness to methacholine may be attenuated in patients using anti-inflammatory therapy and may not be feasible if the FEV_1_ % predicted is very low [36]. Therefore, other less commonly utilized confirmatory methods may be needed to enhance sensitivity for attaining objective confirmation of asthma diagnosis. Excessive variability (>10%) in twice-daily PEF over two weeks was found to be 43% sensitive and 75% specific for asthma [37]. Variation in FEV_1_ of 12% and 200 mL between-visit has good specificity (94%) for diagnosing asthma, but it is not very sensitive (17%) [38]. In some patients with frequent asthma symptoms, stepping-up controller medication and rechecking lung function in 3 months may be warranted to demonstrate this variability [3]. Less sensitive than a methacholine challenge is demonstrating the presence of indirect airway hyperresponsiveness by mannitol, which is no longer available in the USA, or by a dry air exercise challenge, which may be useful among patients with active asthma or to exclude cough-variant asthma [39]. However, it should be recognized that bronchoprovocation tests such as the methacholine challenge are usually performed in a pulmonary function test laboratory, are not readily available in a provider’s office, and can be time-consuming [40]. Such factors can pose additional challenges for utilizing these tests to ensure proper asthma diagnosis.

The utility of FeNO measurement in confirming asthma diagnosis is of increasing interest. Devices that measure the nitric oxide in expired air are becoming increasingly available in many specialty provider offices. The role of FeNO in confirming asthma diagnosis remains controversial. FeNO measurement, which was used by 25% of our providers to confirm asthma, is not recognized as an objective way to confirm asthma diagnosis by the current GINA 2020 guidelines [3,41]. FeNO can be confounded by variables such as cigarette smoking, diet, age, atopy, and corticosteroid use [42,43]. However, the National Institute for Health and Care Excellence lists FeNO as a confirmatory test in patients with symptoms suggestive of asthma [44]. The American Thoracic Society guidelines recognize the use of FeNO in support of asthma diagnosis when objective evidence is lacking [41]. As more clinical data accumulate and a clear consensus from governing societies emerges, the use of FeNO can provide an alternative tool for providers to confirm asthma diagnosis for their patients.

While severe asthma can result in the loss of the reversible nature of airway obstruction [45], patients in our study who lacked confirmatory studies had a higher FEV_1_ % than predicted, indicating less severe disease. Nonetheless, our results indicate that confirming asthma diagnosis before starting biologic modifiers can be especially difficult and can be complicated by a sense of urgency to alleviate patient suffering. Therefore, utilizing other less common confirmatory methods in addition to the bronchodilator response assessment and methacholine challenge can provide an opportunity to enhance adherence to the current guidelines.

In addition to the variability in the confirmatory studies used for asthma diagnosis, our study showed that major comorbidities with the exception of GERD were infrequently addressed. However, the survey results demonstrated that providers do attempt to identify and address these comorbidities. The large discrepancy between the EMR data and the provider-reported survey data may indicate obstacles in addressing these comorbidities. Current GINA guidelines advocate for addressing asthma comorbidities prior to the initiation of biologics. Our survey data showed that the least often addressed asthma comorbidities were obesity, the use of beta blockers and ACE-Is and depression and anxiety. The negative impact of these factors on asthma control may be underappreciated. Obesity is associated with increased incidence of asthma [46] and poor symptom control [47]. The mechanisms behind the effect of obesity on asthma are not fully understood, but proposed processes include lung mechanics such as decreased lung volume [48] and increased systemic inflammation due to pro-inflammatory cytokines released from adipose tissue [49]. Obese individuals are less responsive to ICS than non-obese individuals [50,51], and this is possibly due to modifications in the glucocorticoid response pathways in obese asthmatics [52]. Beta-blockers increase bronchoconstriction [53,54], and while non-selective beta-blockers have a greater effect, selective beta-blockers can also cause bronchoconstriction depending on the dose and individual predisposition [55]. Although ACE-Is are considered to be safe in many asthmatics, cases of ACE-I-induced bronchoconstriction have been reported [56]. Further, an ACE-I-induced cough can confound asthma control. The relationship between asthma and mood disorders such as depression and anxiety is complex. Anxiety and depression occur frequently in asthmatics and they are known risk factors for perception of poor asthma control [57,58]. Treatment of these disorders is associated with improved asthma control [59].

**Figure A1:**
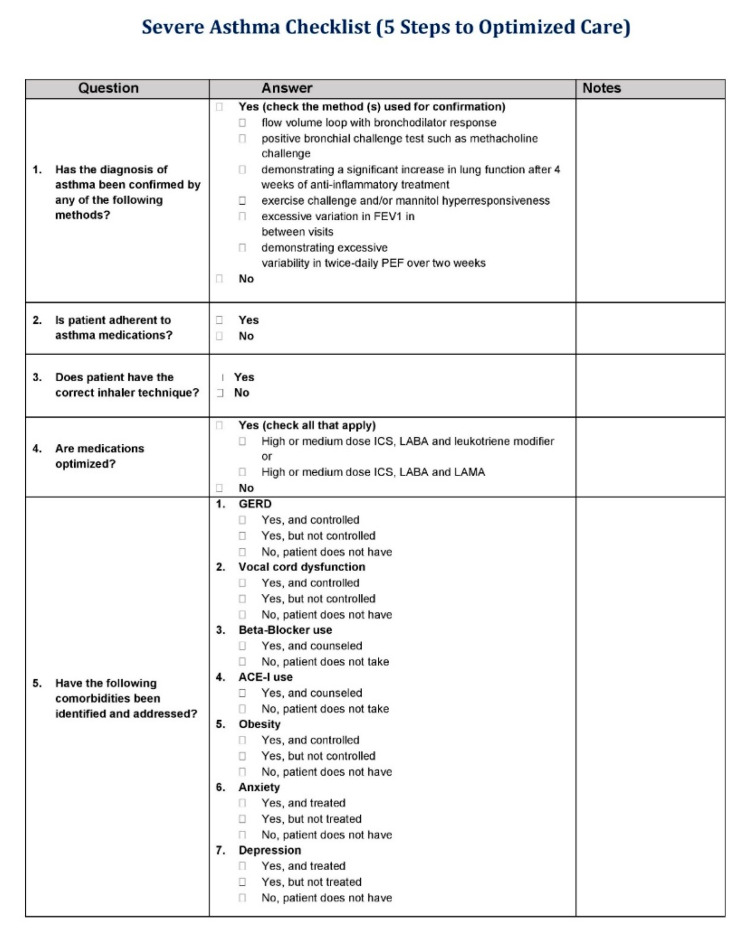
Sample Asthma Checklist.

In assessing compliance with current guidelines, our retrospective study was limited by relying on proper documentation and, therefore, might have resulted in an underestimation of the actual figures. Thus, some of the discrepancies between the EMR and survey data, especially addressing comorbidities, may be accounted for by a lack of appropriate documentation. Additionally, provider response rate to the survey was 80%, which may have also affected the results. Confirming asthma by response to anti-inflammatory therapy over time may be especially difficult to assess in a retrospective record review. Further, it was difficult to objectively determine patient adherence to inhalers, another frequent cause of uncontrolled asthma, and was hence not incorporated in our retrospective chart search. Nonetheless, the study highlighted unmet problems in specialty clinics when approaching patients with difficult-to-treat asthma. Current guidelines do not offer guidance for diagnosing asthma in this population, who represent a unique diagnostic challenge. As indicated by our EMR data and survey data, misconceptions about asthma diagnosis and comorbidities and not remembering to address these factors are the main reasons for inadequate asthma management prior to the initiation of biologics. Therefore, interventions that adopt a systematic approach such as a standardized checklist may aid providers in properly diagnosing asthma and addressing comorbidities. Checklists have been successfully implemented in certain healthcare settings such the intensive care unit to decrease the rate of blood stream infections [60] and in the operating room to decrease procedural errors [61]. Likewise, checklists are used to aid in the diagnosis of certain psychiatric conditions such as depression [62]. However, many other areas of healthcare underutilize checklists, which have been proposed as a way to reduce diagnostic errors [63]. Such interventions may be especially useful in patients with severe uncontrolled asthma prior to considering the use of the costly biologic therapy. A sample checklist applicable to uncontrolled severe asthmatics is shown in Figure A1. Future studies may opt to evaluate the effectiveness of such interventions.

## 5. Conclusions

In this descriptive quality assessment study, we examined the adherence of a group of practicing allergists and immunologists to the current guidelines for the evaluation and management of patients with severe asthma. We also elicited provider habits and perception of these recommendations. We showed that asthma confirmation remains the number one challenge and that comorbidities remain poorly addressed in these patients. These results highlight the gap between current guidelines and everyday practice and prompt for further studies to discover innovative interventions that may enhance adherence and ensure value-based care for these patients.

## Figures and Tables

**Table 1:** Patient Characteristics.

Characteristics	Value ^†^
Age, (years)	55.5 (50–64)
Gender	
Female	26 (65)
Male	14 (35)
Biologic used	
Omalizumab	29 (72.5)
Benralizumab	6 (15)
Mepolizumab	4 (10)
Dupilumab	1 (2.5)
Asthma Therapy Optimized	38 (95)
Asthma Diagnosis Confirmed	23 (58)
BMI (kg/m^2^)	33.8 (28–40)
Obesity	29 (73)
FEV_1_ % predicted	61.5 (47.5–76.5)
Comorbidities addressed	
Gastroesophageal reflux disease (GERD)	28 (70)
Vocal cord dysfunction	10 (25)
Beta-blocker and angiotensin-converting enzyme inhibitor (ACE-I)	0 (0)
Depression and anxiety	18 (45)
Obesity	4 (14)
Inhaler technique	9 (22.5)

^†^ Values are presented as n (%) for categorical variables and median (interquartile range) for continuous variables.
